# Safety Evaluations of Single Dose of the Olive Secoiridoid *S*-(−)-Oleocanthal in Swiss Albino Mice

**DOI:** 10.3390/nu12020314

**Published:** 2020-01-25

**Authors:** Abu Bakar Siddique, Judy Ann King, Sharon A. Meyer, Khaldoun Abdelwahed, Belnaser Busnena, Khalid A. El Sayed

**Affiliations:** 1 Department of Basic Pharmaceutical and Toxicological Sciences, College of Pharmacy, University of Louisiana at Monroe, 1800 Bienville Drive, Monroe, LA 71201, USA; siddiqab@warhawks.ulm.edu (A.B.S.); meyer@ulm.edu (S.A.M.); abdelwks@warhawks.ulm.edu (K.A.); belnaser.busnena@uob.edu.ly (B.B.); 2 Department of Pathology and Translational Pathobiology, LSU Health Shreveport, 1501 Kings Highway, Shreveport, LA 71103, USA; jkin12@lsuhsc.edu

**Keywords:** acute toxicity, extra-virgin olive oil, histopathology, *S*-(−)-oleocanthal, single dose

## Abstract

Epidemiological and clinical studies compellingly showed the ability of Mediterranean diet rich in extra-virgin olive oil (EVOO) to reduce multiple diseases such as cancer, cardiovascular diseases, and aging cognitive functions decline. The *S*-(−)-Oleocanthal (OC) is a minor phenolic secoiridoid exclusively found in extra-virgin olive oil (EVOO). OC recently gained notable research attention due to its excellent in vitro and in vivo biological effects against multiple cancers, inflammations, and Alzheimer’s disease. However, OC safety has not been comprehensively studied yet. This study reports for the first time the detailed safety of oral single OC dose in Swiss albino mice, applying the OECD 420 procedure. Male and female Swiss albino mice (*n* = 10) were orally treated with a single OC dose of either 10, 250, or 500 mg/kg bodyweight or equivalent volumes of distilled water. Mice fed a regular diet, and carefully observed for 14 days. Further, mice were then sacrificed, blood samples, and organs were collected and subjected to hematological, biochemical, and histological examinations. OC 10 mg/kg oral dose appears to be without adverse effects. Further, 250 mg/kg OC, p.o., is suggested as a possible upper dose for preclinical studies in the future.

## 1. Introduction

The Mediterranean diet includes extra-virgin olive oil (EVOO) as a major source of dietary fat, which correlate with its most positive health-promoting outcomes. The daily average intake of EVOO in Mediterranean populations is in the range of 30–50 mL [1,2,3]. The natural occurrence of OC in EVOO ranges from 10 mg/L to 1200 mg/L. This means the average daily consumption of Mediterranean individuals ranges from 0.3/0.5 mg to 36/60 mg OC, depending on the consumed EVOO quality [1,2]. Meanwhile, the high dietary intake of EVOO is considered as a mediator for lowering the incidences of several diseases such as cardiovascular and metabolic diseases, different types of malignancies, Alzheimer’s disease, and osteoporosis [3,4,5,6,7,8,9]. In addition, EVOO phenolic compounds are well known to possess different biological activities including antioxidant, anti-inflammation, anticancer and anti-diabetic activities [10,11,12,13]. EVOO has already shown its important role in human clinical studies via reducing the oxidative stress and inflammation and acute anti-platelet aggregation activities [14,15,16]. 

More than 200 different natural compounds have been identified from olive oil so far, including fatty acids, their triglycerides, sterols, carotenoids, terpenoids, flavonoids, tocopherols, and mono- and polyphenols [17]. Among them, oleic acid has been identified as the most abundant monounsaturated fatty acid in EVOO. However, the beneficial effects of EVOO are mainly associated with the minor olive phenolic components. EVOO polyphenols are minor secondary metabolites that consist of multiple phenolic structural classes [17]. Among these, the olive monophenol secoiridoid *S*-(−)-oleocanthal (OC) is on the top-most scientific attention due to its exceptional and remarkable biological activities in several diseases, even though it makes up only 10% of the EVOO total phenolics content [1,18].

(-)-Oleocanthal has been showing potent anti-inflammatory, antioxidant, antimicrobial, anticancer, and neuroprotective activities. OC exerted its anti-inflammatory effect by concurrently inhibiting COX-1/2 and 5-LOX, which translated to equipotent anti-inflammatory activity to the common non-steroidal anti-inflammatory drug (NSAID) ibuprofen [19]. In addition, OC also inhibited LPS-mediated upregulation of proinflammatory signaling molecules, including interleukin-1β (IL-1β), interleukin-6 (IL-6), macrophage inflammatory protein-1α (MIP-1α), tumor necrosis factor-α (TNF-α), and granulocyte-macrophage-colony-stimulating factor. Recently, OC has emerged as a potential therapeutic agent against neurodegenerative disease, especially in Alzheimer’s disease (AD). In Alzheimer’s disease, OC modulated the structure and function of the β-amyloid by improving the clearance of the amyloid beta protein from neurons. In addition, OC has also shown its prominent activity in promoting degradation of the AD hallmark Aβ and reduced astrocytic inflammatory activation and at the same time, restoring the expression of the neuro-supportive protein in astrocyte and neuroblastoma cells [20,21].

Numerous studies have already shown diverse applications of OC against different cancers both in preventive and/or in treatment modes. OC has shown promising in vitro and in vivo anticancer effects against melanoma [22], breast cancer [10,23,24,25,26], prostate cancer [24], hepatocellular carcinoma [27,28], colon cancer [27,29], multiple myeloma, [30] and leukemia [31]. The activity of OC is highly selective to cancerous cells and exhibits less or no cytotoxic effects on primary or non-tumorigenic cell lines, including human adult dermal fibroblast HDFa cells [32], human mammary epithelial MCF10A cells [10], human liver LO2 cells [28], murine macrophages J774A.1 cells [30], human fibroblast BJ cells [33], rat fibroblast 3Y1 cells [33], human lung fibroblast IMR90 cells [33], and isolated primary human hepatocytes [27]. Though OC showed in vitro high degree of selectivity toward malignant cells, with less or no cytotoxic effect to normal or non-tumorigenic cells yet in vivo preclinical safety and toxicity studies of OC are lacking.

Various doses and route of administration have been used to assess the in vivo bioactivity of OC. Intra-peritoneal 5 mg/kg, 3 X/week and 10 mg/kg daily oral OC treatments significantly suppressed the breast tumor growth, proliferation, and angiogenesis of subcutaneous xenografted triple-negative breast cancer (TNBC) MDA-MB-231 cells and luminal B BT-474 BC cells in athymic nude mice via inhibition of c-MET receptor tyrosine kinase (RTK) [9,34,35,36,37]. OC inhibited the 17β-estradiol-induced proliferation of MCF-7 cells, BT-474 cells, and T-47D cells by interfering with their ER signaling [36]. The combination treatment of OC with tamoxifen (a selective estrogen receptor modulator) or lapatinib synergistically suppressed the proliferation of luminal B type breast cancer cells at in vitro and in vivo studies [34,36]. OC showed potent in vivo breast cancer recurrence inhibition against triple-negative and the luminal B type BC cells [37,38,39]. OC exerted antiproliferative and apoptosis-promoting effects against the HCCLM3 and orthotopic hepatocellular carcinoma in patient-derived xenograft tumor tissues [28]. Interestingly, OC also significantly reduced the lung metastasis and growth from HCCLM3 xenografts [28]. In female BALB/c athymic nude mice, OC (10 mg/kg/day) inhibited A375 xenograft-induced tumor growth, proliferation, and angiogenesis via the activation of caspase-3/9 and the suppression of STAT3, JAK2 and Src kinase signaling [22]. In addition, OC (15 mg/kg/day) also significantly reduced lung metastasis of nude mice that received an A375 xenograft tail vein injection [22], which further confirmed earlier findings of lung metastasis prevention and growth inhibition. In all these studies, OC did not significantly change the bodyweight of nude mice, which suggested preliminary low toxicity profile in animals [10]. Regardless of the pharmacological benefits of the OC, detailed knowledge about acute or sub-acute toxicity of OC is remain unraveled. 

OC dose range used in animal disease models is 5–30 mg/kg, which is difficult to achieve in humans by direct EVOO consumption. Thus, future therapeutic applications of OC will be either as nutraceutical or as prospective investigational new drug (IND) candidate. The former direction is more feasible but hindered due to the lack of preclinical toxicity studies. The literature review clearly indicated that OC might be the most bioactive phenolic constituent in EVOO with the top potential for clinical therapeutic applications. Therefore, the present investigation aimed to investigate the acute single oral and intraperitoneal dose toxicity of OC in Swiss albino mouse model, as an initial step to assess its safety for possible use in human clinical trials.

## 2. Materials and Methods 

### 2.1. Chemicals and Reagents

All reagents purchased from Sigma–Aldrich (St. Louis, MO, USA), unless otherwise stated.

### 2.2. Extraction of (-)-Oleocanthal from Extra-Virgin Olive Oil

OC was extracted from EVOO (The Governor, batch #5-214000-242017). Separation was performed using the previously reported liquid–liquid extraction technology, extracting EVOO with water, resin entrapment, followed by ^1^H NMR-guided size exclusion chromatography on Sephadex LH20, using isocratic dichloromethane elution [35]. Pure OC sample was stored at −20 °C in amber glass vials under N_2_ gas until used for animal dosing [35].

### 2.3. HPLC Analysis

OC purity, >99%, was assessed by using HPLC analysis on a Shimadzu HPLC system equipped with a UV/Visible variable wavelength detector. Briefly, OC was dissolved in 100% CH_3_CN. Samples (20 μL) were then injected onto a Phenomenex Cosmosil 5C18-AR-II column (250 × 4.6 mm, 5 μm; Phenomenex Inc., Torrance, CA, USA). The flow rate of the mobile phase (CH_3_CN-H_2_O 1:1, isocratic) was 1.0 mL/min. Detection was by UV absorbance simultaneously at λ = 230 and 254 nm, with 13.9 min OC retention time. Data acquisition and analysis were accomplished using Lab Solution™ chromatography software [35].

### 2.4. OC Identity and Purity Confirmation by NMR Spectral Analysis

The identity of OC was unambiguously defined by extensive 1D and 2D NMR spectroscopic analyses using a JEOL Eclipse ECS-400 NMR spectrometer. Quantitative ^1^H NMR (q^1^H NMR) and ^13^C NMR analysis were used to assess and confirm OC purity. The OC-rich residue, obtained according to the above-described extraction procedure, was dissolved in 750 μL CDCl_3_ in a 5-mm NMR tube. ^1^H and ^13^C NMR spectra were recorded using tetramethylsilane (TMS) as an internal standard, on a JEOL Eclipse-ECS NMR spectrometer operating at 400 MHz for ^1^H NMR and 100 MHz for ^13^C NMR. A single data set of each of ^1^H and ^13^C-PENDANT NMR experiments confirmed that OC had >99% purity. Typically, 16 scans were collected into 32 K data points over a spectral width of 0−20 ppm with a relaxation delay of 1 s and acquisition time of 2.1 min for the ^1^H NMR spectrum [35].

### 2.5. In Vivo Studies

#### 2.5.1. Animals

Male and female Swiss albino mice (4–5 weeks old) were purchased from Envigo (Indianapolis, IN, USA). The animals were acclimated to the animal housing facility and maintained under clean room conditions in sterile filter-top cages with Alpha-Dri bedding and housed on high efficiency particulate air-filtered ventilated racks at a temperature of 18–25 °C, with a relative humidity of 55–65% and a 12 h light/dark cycle, for at least one week before the study. The mice had free access to drinking water and pelleted rodent chow (no. 7012, Envigo/Teklad, Madison, WI, USA) [40]. The Envigo diet 7012 is autoclavable fixed formula diet designed to support growth and reproduction of rodents. It is enriched with 300–600 mg/kg daidzein and genistein aglycone equivalent isoflavones. Envigo diet 7012 lacks animal protein and fish meal to minimize nitrosamine contents but it is supplemented with additional vitamins including the phenolic tocopherols (vitamin E, 150 IU/kg) to maintain nutritional adequacy after autoclaving [40]. Animals were housed in group cages, each n = 10 animals/experimental group (male, n = 5 and female, n = 5). All animal experiments were approved by the Institutional Animal Care and Use Committee (IACUC), University of Louisiana at Monroe, and were conducted in strict accordance with good animal practice as defined by NIH guidelines (Protocol# 18MAY-KES-01).

#### 2.5.2. *S*-(−)-Oleocanthal Orally Administered Single Dose Acute Toxicity Study in Male and Female Mice

An acute oral toxicity study was performed following the Organization of Economic Co-Operation and Development (OECD) guideline 420 for testing of chemicals with little modification. Mice of both sexes aged 6–8 weeks old, were used. Animals left for one week for adaptation to laboratory conditions before their use in any experiment. To titrate OC maximum tolerated dose, a pilot mice morbidity study has been conducted, *n* = 3, by orally administering the following progressive doses to animals; 10 mg/kg → 50 mg/kg → 100 mg/kg → 250 mg/kg → 500 mg/kg and closely observing animal clinical responses. There was no animal morbidity observed up to 500 mg/kg oral OC single treatment dose neither in male nor in female mice. Thus, mice were then randomly divided into four groups, 10 each, males *n* = 5 and females *n* = 5, for single dose acute toxicity study over 14 days. These groups were as follows: (i) vehicle-treated control group, (ii) OC oral 10 mg/kg treated group, (iii) OC oral 250 mg/kg treated group, and (iv) OC oral 500 mg/kg treated group. Treatments were administered orally after freshly dissolving OC in sterile DMSO/water vehicle. OC 20 mg initially dissolved in 100 µL sterile DMSO as a stock concentration and further diluted with sterile water and each final solution was administered by a ball-tipped plastics gavage needle (70 mm length) at a rate of 20 mL/kg in both sex mice. Animals were carefully observed individually after dosing during the first 4 h, and periodically over the first 24 h, and daily thereafter, for a total of 14 days. All observations were systematically and carefully recorded for each animal. The whole body mass of each animal was determined shortly before dosing. All animals were sacrificed non-fasting at the end of the observation period and subjected to a necropsy.

### 2.6. Data Collection

At the end of the study (14-day of experiment), animals were weighed anesthetized by isoflurane, euthanized by cervical dislocation, decapitated, and dissected to collect blood and harvest internal organs (brain, lung, heart, liver, kidney, spleen, pancreas and small intestine) for biochemical, morphological, and histopathological examination.

### 2.7. Hematological Evaluation

Fresh blood was collected into heparinized Microtainers and transferred to EDTA-containing tubes for hematology. All the blood samples were used free of clots. WBC, RBC, Hgb, Hct, MCV, MCH, MCHC, CHCM, RDW, PLT, MPV and PCT were determined with the Siemans Advia 120 hematology analyzer system (Siemens Healthcare Diagnostics, Inc., Tarrytown, NY, USA) at the facility of LSU-School of Veterinary Medicine (SVM) Clinical Pathology Laboratory. Biochemical analysis of heparinized plasma included determination of glucose, BUN, creatinine, AST, ALP, ALT (SA), GGT (LA) with the Beckman AU680 clinical chemistry analyzer system (Beckman Coulter, Atlanta, USA) at the LSU-SVM Clinical Pathology Laboratory.

### 2.8. Hematoxylin and Eosin Y (H&E) Staining

Different organ tissues were freshly collected and immediately fixed in 10% neutral buffered formalin for 48 h. The tissues were further transferred to 70% ethanol, processed, and embedded in paraffin. All the sectioning and H&E staining has been done at the AML Laboratories (Jacksonville, FL, USA). Briefly, paraffin-embedded tissues were sliced into 5 µm sections and mounted on positively charged slides, dewaxed with xylene, rinsed with alcohol, rehydrated by water, and finally, the tissue slides were stained with H&E. Tissues were then dehydrated through ethanol to xylene and coverslipped with Permount [37].

### 2.9. Statistical Analysis

All values were expressed as the mean ±SD (standard deviation) and the results were analyzed statistically by one-way Analysis of Variance (ANOVA) followed by Tukey’s tests using statistical software-GraphPad Prism (GraphPad Software, Inc., La Jolla, CA, USA) version 8.0. *p* < 0.05 compared to control considered to be statistically significant.

### 2.10. In Vivo Up-and-Down Procedure for LD_50_ Determination

In vivo Up-and-Down Procedure (UDP) using Swiss albino mice used to determine OC LD_50_. The UDP is a newer alternative method to the fixed-dose protocol for estimating LD_50_, with the use of fewer animals [41,42,43,44]. The endpoint monitored was the mice survival or morbidity upon dosing [41,42,43]. The therapeutic dose we previously used in breast cancer model was 5 mg/kg, ip, 3 X/week [10]. Thus, this dose was used as a starting point. Half-log increments or reductions were then applied for subsequent doses based on animal response with a slope factor σ 0.33 and a starting dose of 16 mg/kg, ip (3.2 X reported OC therapeutic dose, 5 mg/kg). Each subsequent ip dose was determined based on the response (survival or morbidity within 24 h) observed from the previous dose. The OC ip doses utilized in the study were 16.00, 51.20, 163.84, and 524.29 mg/kg. OC treatments were prepared by dissolving in sterile normal saline just prior to the injection. Dose progression was stopped when 1/3 stopping rules of AOT425StatPgm program was satisfied [41,42]. Qualitative appearance of righting reflex, forelimb strength and incidence of any tremors and/or convulsions were be made at 10–15 min intervals over 1st 8 h and daily thereafter over the 14-day observation period. Mice were weighed at the beginning of the study and then at 7 and 14 days. Calculated ip LD_50_ of OC was in 164–524 mg/kg range.

## 3. Results

### 3.1. Preliminary Clinical Observations after OC Single Dose Administration in Male and Female Swiss Albino Mice 

A single oral dose administration of 10, 250, and 500 mg/kg OC produced no gross adverse and/or behavioral responses in both male and female Swiss albino mice groups over the 14 days observation period compared to vehicle control. No deaths or obvious clinical toxicity signs were recorded in any groups throughout the study. None of the mice showed signs of toxicity on their skin, fur, or eyes. No salivation or diarrhea were observed. 

### 3.2. Effect of OC Single Oral Dose on Swiss Albino Mouse Bodyweights 

Mice bodyweight was monitored on the 1st, 7th, and 14th days of the study for all groups including (i) vehicle control-treated group, (ii) 10 mg/kg OC-treated group, (iii) 250 mg/kg OC-treated group, and (iv) 500 mg/kg OC-treated group. No significant changes in the bodyweight observed for OC treatments over the 14 days study course in comparison to the vehicle control group (Table 1).

### 3.3. Effect of OC Single Oral Dose on Swiss Mouse Organ Weights

Organ weights were recorded after all mouse groups sacrificed. There were no significant differences observed in the mice organ weights after the used OC single oral dose over the 14 days experiment in comparison to the vehicle control group (Table 1).

### 3.4. Evaluation of Effects of OC Treatments on Haematological Parameters

The effects of OC at a single dose of 10, 250, and 500 mg/kg or vehicle control on haematological parameters were evaluated (Table 2). Most blood parameters, like total red blood cells (RBCs), total white blood cells (WBCs), and platelet (Plt) counts, mean platelet volume (MPV) and plateletcrit (Pct) in treated mice were not significantly different from the vehicle control treatment (Table 2). Most hematological parameters including haemoglobin (Hgb), Hct, mean corpuscular volume (MCV), mean corpuscular haemoglobin (MCH), mean corpuscular haemoglobin concentration (MCHC), cell hemoglobin concentration mean (CHCM), and red blood cell distribution unit (RDW) in treated mice were not significantly different from the vehicle control treatment (Table 2). However, in male OC 10 mg/kg group, there was slightly decreased RBC and hemoglobin levels in the comparison with the vehicle control group. Mice of the higher OC doses, 250 and 500 mg/kg, showed higher levels of platelet count, Pct, and RWD, compared to vehicle control-treated group in both male and female mice. This elevation still does not imply severe hematological toxicity. 

### 3.5. Biochemical Analysis of Mice Sera 

The effects of OC single dose treatments of 10, 250, and 500 mg/kg on Swiss albino mice serum biochemical parameters was compared to vehicle control treatment (Table 3). The kidney function parameter, creatinine, did not reveal any significant changes but blood urea nitrogen (BUN) level was significantly decreased in treated male groups. Meanwhile, the OC 250 and 500 mg/kg-treated female mice showed significant increase in the BUN level compared to respective vehicle control group. No statistically significant differences in the liver function parameters like alanine aminotranferase (ALT), aspartate aminotransferase (AST), and alkaline phosphatase (ALP) were observed in female OC-treated mice except the OC 10 mg/kg and 500 mg/kg groups, which showed AST level decrease. All OC treatments in male groups showed significant ALT level decrease and only the 500 mg/kg female mice showed significant ALT level decrease versus the vehicle control-treated groups. Similarly, all treated groups showed significantly reduced level of ALP. Additionally, the mice serum glucose level in all treated groups was significantly increased in both male and female treated groups compared to the vehicle control group (Table 3).

### 3.6. Histopathological Evaluation of the Effects of OC Dosing on Mice Brain, Heart, Lung, Liver, Kidney, Spleen, Pancrease, and Small Intestine

Microscopical examination of H&E stained sections of 10–500 mg/kg OC-treated mice brain, lung, liver, spleen, pancreas and small intestine showed mostly normal histology versus vehicle-treated controls (Supplementary Figures S1–S6). Additionally, macroscopic examination of OC-treated mice organs revealed no abnormalities in the color or texture compared to the vehicle control treated group. One feature noted was the extramedullary hematopoiesis in spleen, as typical for Swiss albino mice, that appeared to be of comparable frequency in both vehicle and OC-treated mice. There were observable intra-alveolar blood and thickened septa in all lung sections but most likely these are post-mortem artifacts. No additional lung lesions that evidently differentiated vehicle and OC-treated mice.

**Figure 1:**
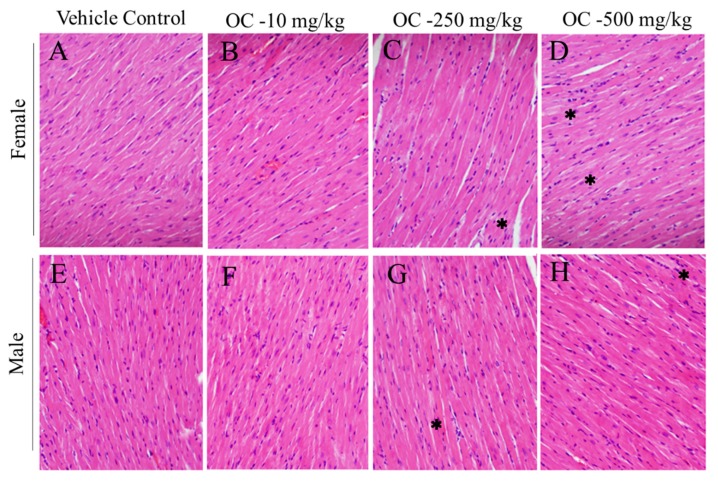
Histopathological images of Swiss albino mice heart tissue sections after 14 days of exposure to single OC dose treatments. (**A**) Female vehicle control, (**B**) Female OC-10 mg/kg, (**C**) Female OC-250 mg/kg, (**D**) Female OC-500 mg/kg, (**E**) Male vehicle control, (**F**) Male OC-10 mg/kg, (**G**) Male OC-250 mg/kg, (**H**) Male OC-500 mg/kg representative sections. * Focal hypochromasia.

Higher treatment doses of OC, 250 and 500 mg/kg, exhibited a tinctural change in the H&E stained heart sections, with visible hypochromic foci within the characteristic eosinophilic alignment of cardiomyocytes. The pattern of foci appeared random and frequency was somewhat dose-dependent in both male and female mice (Figure 1). The therapeutic dose OC, 10 mg/kg, was without this pattern.

**Figure 2:**
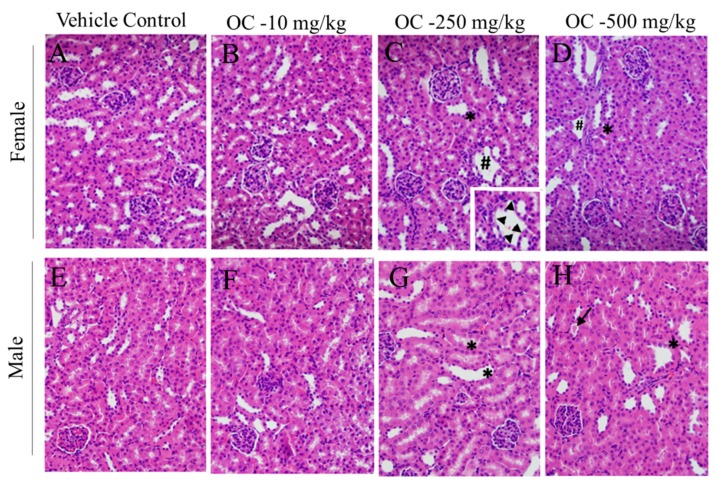
Histopathological images of Swiss albino mice kidney sections 14 days after exposure to OC single dose treatments. (**A**) Female vehicle control, (**B**) Female OC-10 mg/kg, (**C**) Female OC-250 mg/kg, (**D**) Female OC-500 mg/kg, (**E**) Male vehicle control, (**F**) Male OC-10 mg/kg, (**G**) Male OC-250 mg/kg, (**H**) Male OC-500 mg/kg representative sections. * tubular dilation, #tubular dropout.

Kidneys of the mice treated with higher doses of OC appeared to be the most adversely affected organ. Dilation of renal distal tubules was frequent in kidney sections from male and female mice treated with OC 250 and 500 mg/kg. Tubular dropout was observed in micrographs from female mice treated with these OC high doses (Figure 2). A small tubular cast was observed in one OC 500 mg/kg treated male mouse. Glomeruli appeared normal for all groups. Mice treated with OC therapeutic dose, 10 mg/kg, did not show this kidney toxicity pattern.

The frequency and nature of structural changes observed in male and female mice kidney and heart micrographs across all OC doses are summarized in Supplementary Table S1.

### 3.7. Assessment of Acute i.p. Oleocanthal Toxicity with the Up-and-Down Procedure

The UDP OC toxicity in Swiss albino male and female mice using 16.00, 51.20, 163.84, and 524.29 mg/kg OC ip treatment doses. This experiment estimated the LD_50_ of OC in the range of 164–524 mg/kg.

## 4. Discussion

Comprehensive evaluation of the toxic characteristics of a molecule is usually a preliminary step to assess the safety of prospective new drug candidates and even natural product nutraceuticals [44]. Safety evaluation process involves preliminary determination of LD_50_ in animal model as an initial step before subsequent studies. The acute toxicity study may provide initial important information on the toxic mode of action and target organ(s) of tested lead, which can be used as a foundation for risk classification to establish tolerable safe therapeutic doses [45,46]. Although OC has several favorable pharmacological and health benefits, its safety cannot be automatically assumed based on its long-term human intake in EVOO. This is mainly because OC natural occurrence in EVOO can range from 10 mg to 1200 mg/L and the 20 mL daily EVOO consumption translate to 0.2–24 mg daily OC consumption, which is still far from therapeutic doses used in animal models.

Most of the OC health beneficial effects are derived from cellular studies and, to a limited extent, animal studies [10,28,34,35,36]. Translating the results from cell culture studies to humans can be misleading because the concentrations used might not be realistically and physiologically achievable. Unlike many bioactive natural products, OC also shows modest in vitro activity but in different mouse models it was far much more potent, suggesting potential in vivo bioactivation, which complicates safety predications. 

In addition, it is also difficult to directly extrapolate the beneficial effects of OC in animal models to humans due to interspecies variations of metabolic and CYP450 isoforms, pharmacokinetics, and pharmacodynamics. Comprehensive knowledge of OC oral toxicity is urgently needed to facilitate establishing its therapeutic and clinical applications. Thus, the present study assesses OC single oral dose acute toxicity in Swiss albino mouse model following the OECD guidelines 420 setting the steppingstone to determine OC oral safety and therapeutic margin [47,48]. Mice rather than rats were used in this study because the mice lethal dose data is established predictors for toxicity in humans [49]. The current study highlights the need for additional studies in different animal models to develop more comprehensive knowledge of OC safety.

The highest used OC oral dose, 500 mg/kg, caused neither animal fatalities nor significant changes in behavioral and clinical patterns, except heart and kidney in both mice genders. Over the 14-days toxicity evaluation course, there was no significant bodyweight variations observed (Table 1). Overall safety of the OC 10 mg/kg dose is consistent with the positive therapeutic outcomes in multiple literature cancer animal studies [10,28,34,35,36]. 

Treated mice serum glucose levels in all groups were significantly increased in both male and female mice, compared to vehicle control. Since blood samples were collected after sacrifice while animals non-fasting, the increased serum glucose level of OC-treated mice may be attributed to enhanced animal food intake capacity. 

Brain, heart, liver, kidney, spleen, and small intestine are the main target body organs that can be affected by any toxic xenobiotics [50,51]. Statistically, no observed significant variations in these organs in treatments group when compared with vehicle control group (Table 1). Micrographs of H&E-stained brain, lung, liver, spleen, pancreases, and small intestine sections showed mostly normal histology, further confirming the potential safety of OC treatments toward these organs in this animal model (Figure 1 and Figure 2 and Supplementary Figures S1–S6). 

The most notable histopathological differences from vehicle-treated mice were for OC highest treatment doses in kidney (Figure 2). Several sporadic lesions were observed, which suggested potential to become pathologically significant. These included renal tubular dilation, tubular cast formation, and tubular dropout. However, the extent of tissue damage was not associated with elevated serum biomarkers of renal toxicity, i.e., elevated creatinine and blood urea nitrogen.

Another observation of pathological concern was the focal hypochromasia in cardiac muscle (Figure 1). Since Swiss mice are prone to amyloidosis with depositions in heart, it is reasonable to consider amyloid deposits pathological and as causal to the observed hypochromatic foci. Alternately, a physiological etiology could be glycogen deposits, known to stain poorly with H&E. These conditions can be distinguished by specific stains, Congo Red for amyloid and diastase sensitive-periodic acid/Schiff base for glycogen, which should be employed in follow-up OC toxicity studies. Minor cardiotoxicity observed for all doses (Figure 1). No observed gender-based associated toxicity variations. Overall, high OC doses, 500 mg/kg and above, appears to have the potential to cause cardiomyopathy.

Comparison of the results of OC treatments hematological parameters with those of the control-treated groups showed no toxicity to the haemopoietic system as indicated by the lack of significant parameters changes (Table 2). Additionally, the blood creatinine and BUN levels were not affected (Table 3). ALP is considered as the standard marker of biliary tract obstruction [52]. In this study, OC-treated groups at all doses showed significant decrease in ALP, AST, and ALT levels (Table 3), which may be indicative of OC hepatoprotective and potential positive effects on liver functions [52]. 

Hematological parameters are sensitive in vivo markers for prediction of the physiological changes in response to xenobiotic intake, environmental pollutant exposure or toxicity stress [47]. Blood platelets have a vital role in blood coagulation. OC-rich EVOO proved to have a potent anti-platelet aggregation in human subjects [14]. Blood samples collected before and 2 h after the intake of the OC-rich EVOO to assess its acute anti-platelet aggregation effects [14]. Platelet aggregation was reported to be dependent on platelet count in human coronary artery disease patients [14]. OC single dosing to Swiss albino mice in this study showed remarkable elevated levels of platelet count in blood collected 14 days after dosing, indicating potential for thrombotic activity (Table 2) [53]. Additional studies are needed to study the effect of OC intake of single dose on platelets aggregation in different models.

The UPD procedure toxicity scheme in Swiss albino mice suggested an ip LD_50_ range of 164–524 mg/kg for OC. This could suggest possible intestinal and/or intestinal microbiota role in minimizing OC oral toxicity since mice tolerated the maximally administered oral dose of 500 mg/kg without morbidity. 

Therefore, this study indicates that the oral administration of OC 10, 250, and 500 mg/kg into the Swiss albino mice showed no mortality, indicating that oral OC LD_50_ value is greater than 500 mg/kg, unlike the ip administration, which showed an LD_50_ range of 164–524 mg/kg in this animal species. This clearly shows the improved OC safety for oral versus parenteral administration. Thus, per the OECD classification, OC can be assigned as a class-4 natural product (LD_50_ > 300–2000 mg/kg). Although the species and inter-individual sensitivity will significantly vary and results extension should only be applicable after careful clinical testing, hypothetical assumption of the oral OC LD_50_ of 500 mg/kg in Swiss albino mice is to be applicable to humans, with the average human bodyweight of 70 kg, knowing that OC average natural occurrence in EVOO range from 10–1200 mg/L, the single dose toxicity of OC tentatively translate to 35 g of pure OC or 29.2–3500 L of EVOO. 

## 5. Conclusions

Grossly, OC 10 mg/kg oral dose was safe and showed no toxicity on any of the investigated Swiss albino mouse organs. Histopathological evidence suggests that at higher doses modest adverse effects on heart and kidney in both male and female Swiss albino mice. OC dosing may have enhanced animals’ food intake and liver functions. OC can be classified as a fourth-class compound in terms of the European Union classification system (EU, Council Directive 92/32/EEC). This suggests OC low toxicity potential and relatively optimal safety in Swiss albino mice at therapeutic dosing level. Additional species-specific studies are needed including the safety assessments in different animal models and its effects on reproductive systems, pregnant animals, and their fetuses. In addition, OC long-term chronic and subacute safety evaluations are also required to set the stage for its future use in clinical trials even as an over-the-counter nutraceutical. 

## Figures and Tables

**Table 1:** The mean (±SD) body and organ weights of Swiss albino mice at the study end.

Index	Male				Female			
	Vehicle Control	OC-10 mg/kg	OC-250 mg/kg	OC-500 mg/kg	Vehicle Control	OC-10 mg/kg	OC-250 mg/kg	OC-500 mg/kg
Body wt. (g)	29.4 ± 1.5	36.6 ± 1.9	32.2 ± 1.8	34.6 ± 2.3	26.0 ± 0.9	28.0 ± 1.8	27.9 ± 2.5	28.6 ± 2.0
Brain (g)	0.5 ± 0.03	0.5 ± 0.04	0.5 ± 0.03	0.5 ± 0.03	0.4 ± 0.04	0.5 ± 0.02	0.4 ± 0.05	0.4 ± 0.06
Heart (g)	0.2 ± 0.01	0.2 ± 0.02	0.2 ± 0.02	0.2 ± 0.02	0.1 ± 0.01	0.1 ± 0.02	0.1 ± 0.02	0.1 ± 0.02
Lung (g)	0.3 ± 0.06	0.3 ± 0.06	0.3 ± 0.03	0.3 ± 0.06	0.2 ± 0.04	0.2 ± 0.04	0.2 ± 0.06	0.2 ± 0.07
Liver (g)	1.0 ± 0.14	1.6 ± 0.07	1.2 ± 0.19	1.5 ± 0.11	1.0 ± 0.12	1.2 ± 0.17	1.2 ± 0.05	1.2 ± 0.13
Spleen (g)	0.06 ± 0.01	0.1 ± 0.01	0.1 ± 0.01	0.1 ± 0.02	0.1 ± 0.02	0.1 ± 0.02	0.1 ± 0.01	0.1 ± 0.01
Kidney (g)	0.5 ± 0.05	0.6 ± 0.03	0.6 ± 0.04	0.6 ± 0.09	0.4 ± 0.02	0.4 ± 0.04	0.4 ± 0.04	0.4 ± 0.03

**Table 2:** Hematology analysis results (mean ± SD) for Swiss albino mice at the study end.

Blood Index	Male					Female		
	Vehicle Control	OC-10 mg/kg	OC-250 mg/kg	OC-500 mg/kg	Vehicle Control	OC-10 mg/kg	OC-250 mg/kg	OC-500 mg/kg
WBC (10^3^/uL)	2.18 ± 0.84	2.86 ± 0.60	3.46 ± 1.78	2.66 ± 1.59	3.48 ± 1.15	3.46 ± 1.09	3.28 ± 0.80	2.74 ± 1.22
RBC (10^6^/uL)	10.10 ± 0.36	8.66 ± 0.53 *	10.02 ± 0.68	9.23 ± 0.40	9.74 ± 0.57	9.56 ± 0.31	9.46 ± 0.17	8.99 ± 0.39
Plt (10^3^/uL)	652 ± 184.8	815 ± 315.9	1007 ± 130.9	910 ± 249.1	440 ± 90.6	501 ± 51.9	338 ± 51.9	928 ± 212.3 *
MPV (fL)	8.32 ± 0.53	8.63 ± 2.13	7.22 ± 0.17	7.38 ± 0.55	7.85 ± 0.34	7.76 ± 0.19	7.90 ± 0.19	7.74 ± 0.48
Pct (%)	0.54 ± 0.13	0.50 ± 0.23	0.72 ± 0.07	0.66 ± 0.13	0.32 ± 0.08	0.41 ± 0.04	0.27 ± 0.04	0.72 ± 0.20 *
Hgb (g/dL)	14.20 ± 0.65	12.36 ± 0.63 *	13.52 ± 0.96	13.12 ± 0.47	14.00 ± 0.56	13.84 ± 0.34	13.40 ± 0.25	13.54 ± 0.43
Hct (%)	44.04 ± 1.92	40.26 ± 3.19	44.78 ± 3.34	43.14 ± 1.63	44.80 ± 2.14	43.90 ± 1.20	43.38 ± 1.20	43.18 ± 2.31
MCV (fL)	44.60 ± 1.02	46.48 ± 1.39	44.70 ± 1.28	46.80 ± 0.97	45.98 ± 0.75	45.92 ± 1.30	45.88 ± 1.30	47.94 ± 0.77
MCH (pg)	14.06 ± 0.34	14.26 ± 0.25	13.54 ± 0.21	14.20 ± 0.39	14.35 ± 0.32	14.46 ± 0.45	14.38 ± 0.45	14.98 ± 0.32
MCHC (g/dL)	31.52 ± 0.32	30.70 ± 1.05	30.28 ± 0.47	30.34 ± 0.30	31.25 ± 0.36	31.54 ± 0.46	30.95 ± 0.46	31.40 ± 1.23
CHCM (g/dL)	30.14 ± 0.30	28.50 ± 0.55	29.24 ± 0.61	28.84 ± 0.36	29.50 ± 0.42	29.50 ± 0.48	28.68 ± 0.48	28.20 ± 0.70
RDW (%)	13.52 ± 0.20	13.56 ± 0.33	13.38 ± 0.24	14.86 ± 0.64 *	13.65 ± 0.17	13.78 ± 0.54	14.20 ± 0.55	14.78 ± 0.61 *

* Indicate statistically significant difference at *p* < 0.05.

**Table 3:** Serum biochemical results of vehicle control and treated Swiss albino mice (mean ± SD).

Blood Index	Male					Female		
	Vehicle Control	OC-10 mg/kg	OC-250 mg/kg	OC-500 mg/kg	Vehicle Control	OC-10 mg/kg	OC-250 mg/kg	OC-500 mg/kg
GLU (mg/dL)	95.0 ± 10.0	240.5 ± 4.5 *	236.0 ± 27.0 *	185.5 ± 12.5 *	205.0 ± 1.0	235.0 ± 1.0	205.0 ± 11.0	274.0 ± 28.5 *
AST (U/L)	173.5 ± 15.5	165.0 ± 76.0 *	93.5 ± 17.5 *	100.0 ± 6.0 *	158.5 ± 10.5	128.0 ± 7.0 *	174.5 ± 0.0	145.5 ± 13.5
ALT (U/L)	62.0 ± 2.0	35.5 ± 12.5 *	25.0 ± 7.0 *	33.0 ± 8.0 *	53.0 ± 14.0	36.5 ± 1.5	41.0 ± 5.0	32.5 ± 2.5 *
ALP (U/L)	15.0 ± 3.0	6.0 ± 1.0 *	5.5 ± 0.5 *	7.0 ± 1.0 *	7.5 ± 2.5	<5.0#	7.5 ± 2.5	7.5 ± 2.5
BUN (mg/dL)	36.0 ± 5.0	21.5 ± 0.5 *	22.0 ± 1.0 *	21.5 ± 0.5 *	21.5 ± 1.5	23.0 ± 0.0	26.0 ± 1.0 *	24.5 ± 1.5 *
CREAT (mg/dL)	<0.2#	<0.2#	<0.2#	<0.2#	<0.2#	<0.2#	<0.2#	<0.2#

* Indicate statistically significant difference at *p* < 0.05, (#) < dL = detection limit.

## Supplementary Materials

The following are available online at https://www.mdpi.com/2072-6643/12/2/314/s1. Table S1: Summary of different organ histopathological evaluations of OC and vehicle control-treated female and male Swiss albino mice. Figure S1: Histopathological images of Swiss albino mice brain sections 14 days after exposure to oleocanthal single dose. Figure S2: Histopathological images of lung tissue from Swiss albino mice 14 days after exposure to oleocanthal single dose. Figure S3: Histopathological images of liver tissue from Swiss albino mice 14 days after exposure to oleocanthal single dose. Figure S4: Histopathological images of intestine tissue from Swiss albino mice 14 days after exposure to oleocanthal single dose. Figure S5: Histopathological images of pancreases tissue from Swiss albino mice 14 days after exposure to oleocanthal single dose. Figure S6: Photomicrographs of H&E stained spleen from Swiss albino mice 14 days after exposure to oleocanthal single dose.
