# Supplementary material for: Safety Evaluations of Single Dose of the Olive Secoiridoid S-(−)-Oleocanthal in Swiss Albino Mice
Source: Nutrients. 2020 Jan 25;12(2):314. doi: 10.3390/nu12020314 (PMC7071127; doi:10.3390/nu12020314)
Supplement: Supplementary file 1 [file nutrients-12-00314-s001.pdf]

nutrients

Supporting information

Safety Evaluations of Oral Single Dose of The Olive Secoiridoid *S*-(-)-Oleocanthal in Swiss albino Mice

Abu Bakar Siddique[1], Judy Ann King[2], Sharon A Meyer[1], Khaldoun Abdelwahed[1], Belnaser Busnena,[1] and Khalid El Sayed[1*]

[1] Department of Basic Pharmaceutical and Toxicological Sciences, College of Pharmacy, University of Louisiana at Monroe, 1800 Bienville Drive, Monroe, Louisiana, siddiqab@warhawks.ulm.edu (A.B.S); meyer@ulm.edu (S.A.M.); abdelwks@warhawks.ulm.edu (A.K.); belnaser.busnena@uob.edu.ly (B.B.); elsayed@ulm.edu (K.E.S.)

[2] LSU Health Shreveport, Department of Pathology and Translational Pathobiology, 1501 Kings Highway, Shreveport, Louisiana, jkin12@lsuhsc.edu

* Correspondence: elsayed@ulm.edu, Tel.: +1318-342-1725

Received: 25 December 2019; Accepted: 20 January 2020; Published: 25 January 2020

nutrients

MDPI

Supplementary Table S1. Summary of different organ histopathological evaluations and tentative toxicity scores of OC and vehicle control-treated Swiss albino mice.

Tissue	Sex	OC Dose (mg/kg)	Toxicity scores (n=5)	Comments
Kidney	Female	Vehicle Control	1,1,1,1,2	1/5 with sporadic hypochromic distal tubules
		10 mg/kg	1,1,1,1,1	0/5 with finding
		250 mg/kg	1,1,1,2	1/4* with tubular dropout
		500 mg/kg	1,1,2,1,2	1/5 with tubular dropout
	Male	Vehicle Control	1,1,1,1,2	1/5 with sporadic exfoliation of proximal tubule cells
		10 mg/kg	1,1,1,2,1	1/5 with sporadic exfoliation of proximal tubule cells
		250 mg/kg	1,2,2,2,2	2/5 with dilated distal tubules<br>1/5 with sporadic exfoliation of proximal tubule cells<br>1/5 with tubular dropout<br>1/5 with mitotic cell
		500 mg/kg	2,3,3,2,2	4/5 with dilated tubules<br>3/5 with vacuolated tubular cells<br>2/5 with sporadic exfoliation of tubular cells<br>1/5 with intratubular cast
Heart	Female	Vehicle Control	1,1,1,1,1	0/5 with finding
		10 mg/kg	1,1,2,1,1	1/5 with hypochromatic foci
		250 mg/kg	2,2,3,2	3/4* with hypochromatic foci
		500 mg/kg	1,2,1,2,2	3/5 with hypochromatic foci
	Male	Vehicle Control	1,1,2,1,1	1/5 with hypochromatic foci
		10 mg/kg	1,1,1,2,1	1/5 with hypochromatic foci
		250 mg/kg	2,1,2,1,1	2/5 with hypochromatic foci
		500 mg/kg	1,1,1,2,3	3/5 with hypochromatic foci

Toxicity result scores: 1 = normal tissue, minimal pathological changes.

2 = modest pathological changes (limited alteration of specialized tissue structure and regularity, e.g., reduced white pulp, macrophage infiltration, apoptosis; red pulp hemosiderin, increased EMG; swelling; vacuolization). 3 = significant pathological changes (necrosis, hemorrhage, extensive inflammatory infiltrate, pyknosis, karyolysis, karyorrhexis).

nutrients MDPI

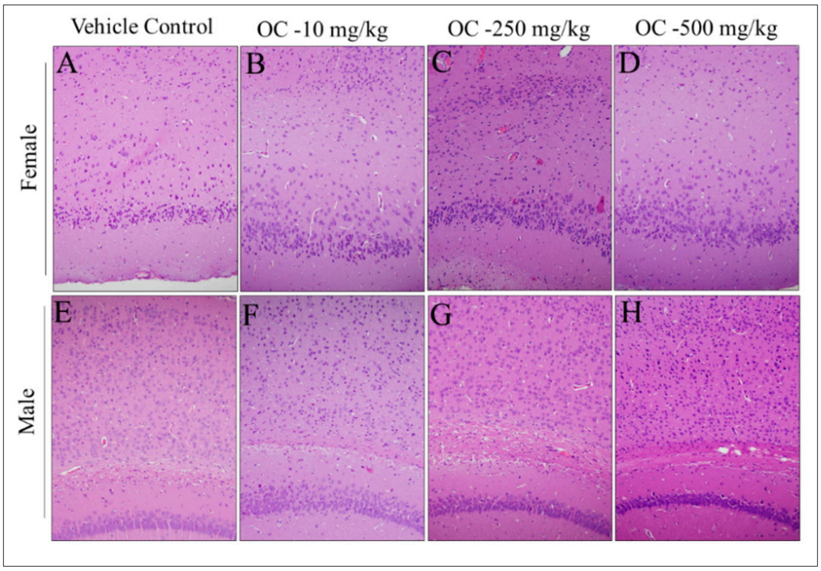

Supplementary Figure S1- Histopathological images of the Swiss albino mice brain sections 14 days after exposure to OC single dose treatments. (A) Female vehicle control, (B) Female OC-10 mg/kg, (C) Female OC-250 mg/kg, (D) Female OC-500 mg/kg, (E) Male vehicle control, (F) Male OC-10 mg/kg, (G) Male OC-250 mg/kg, (H) Male OC-500 mg/kg representative sections.

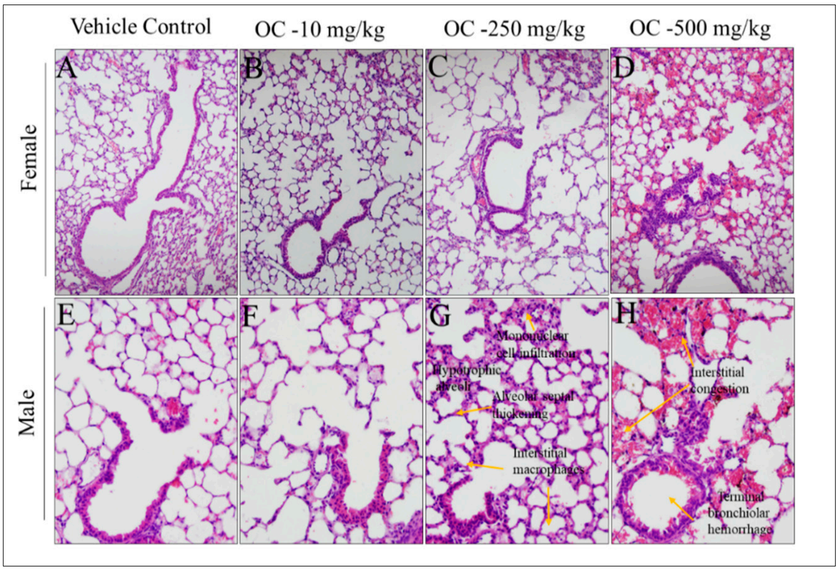

Supplementary Figure S2- Histopathological images of lung sections from Swiss albino mice 14 days after exposure to OC single dose treatments. (A) Female vehicle control, (B) Female OC-10 mg/kg, (C) Female OC-250 mg/kg, (D) Female OC-500 mg/kg, (E) Male vehicle control, (F) Male OC-10 mg/kg, (G) Male OC-250 mg/kg, (H) Male OC-500 mg/kg representative sections.

nutrients

MDPI

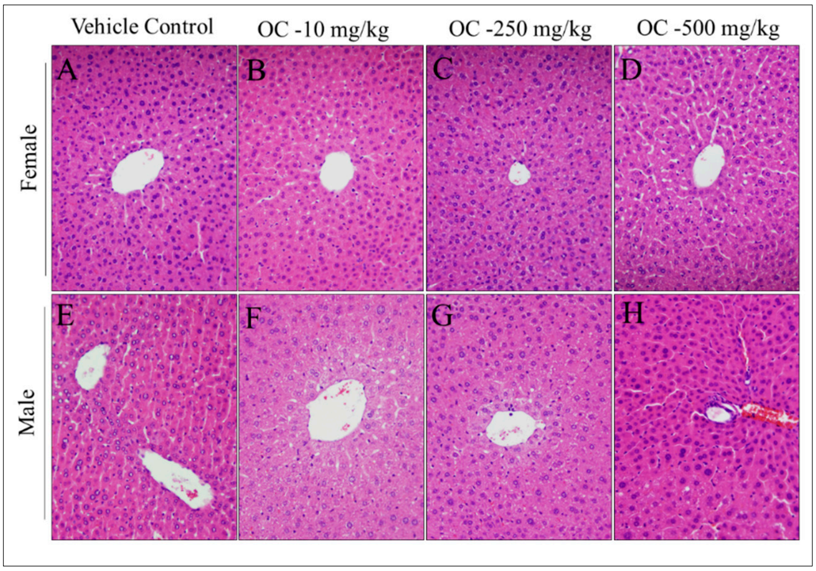

Supplementary Figure S3- Histopathological images of liver tissue from Swiss albino mice 14 days after exposure to OC single dose treatments. (A) Female vehicle control, (B) Female OC-10 mg/kg, (C) Female OC-250 mg/kg, (D) Female OC-500 mg/kg, (E) Male vehicle control, (F) Male OC-10 mg/kg, (G) Male OC-250 mg/kg, (H) Male OC-500 mg/kg representative sections.

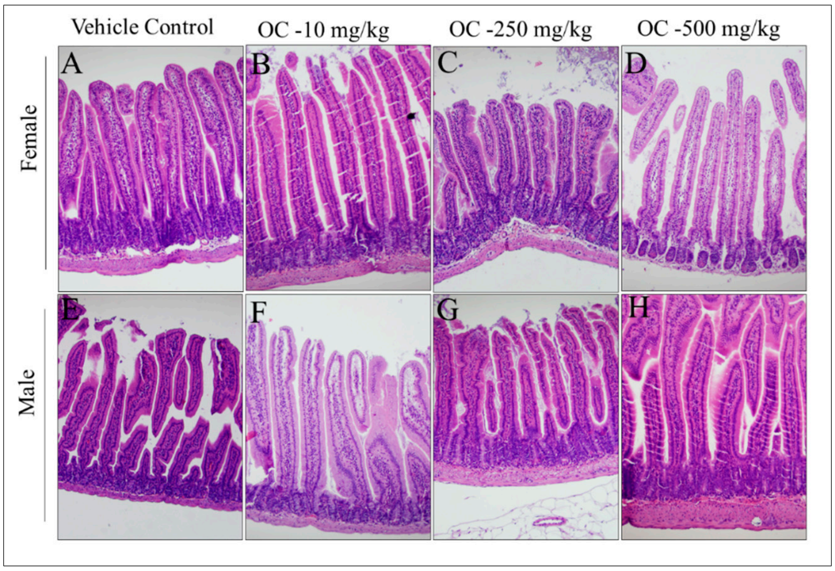

Supplementary Figure S4- Histopathological images of intestine tissue from Swiss albino mice 14 days after exposure to OC single dose treatments. (A) Female vehicle control, (B) Female OC-10 mg/kg, (C) Female OC-250 mg/kg, (D) Female OC-500 mg/kg, (E) Male vehicle control, (F) Male OC-10 mg/kg, (G) Male OC-250 mg/kg, (H) Male OC-500 mg/kg representative sections.

nutrients

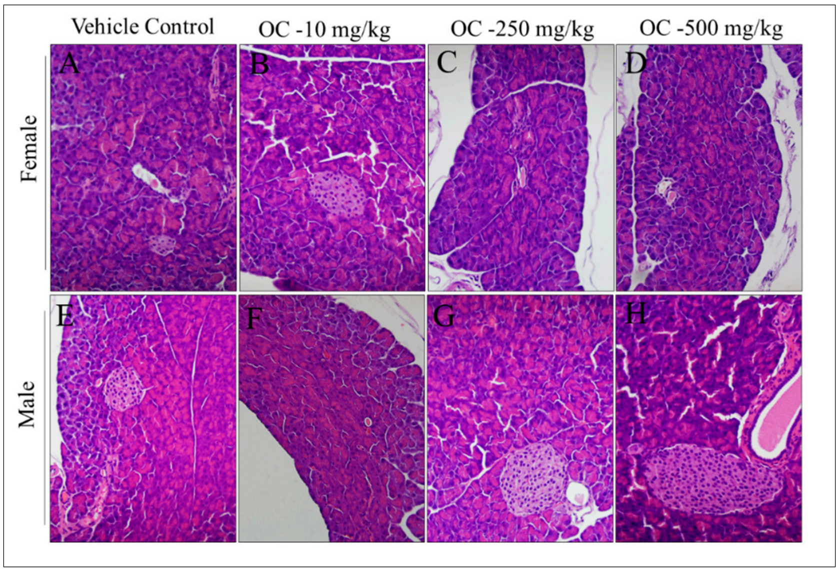

Supplementary Figure S5- Histopathological images of pancrease tissue from Swiss albino mice 14 days after exposure to OC single dose treatments. (A) Female vehicle control, (B) Female OC-10 mg/kg, (C) Female OC-250 mg/kg, (D) Female OC-500 mg/kg, (E) Male vehicle control, (F) Male OC-10 mg/kg, (G) Male OC-250 mg/kg, (H) Male OC-500 mg/kg representative sections.

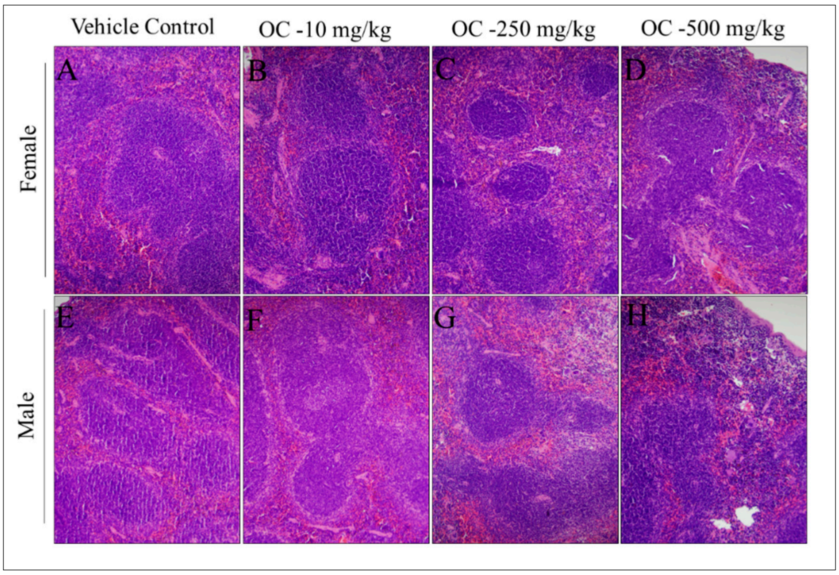

Supplementary Figure S6- Photomicrographs of H&E stained spleen from Swiss albino mice 14 days after exposure to OC single dose treatments. (A) Female vehicle control, (B) Female OC-10 mg/kg, (C) Female OC-250 mg/kg, (D) Female OC-500 mg/kg, (E) Male vehicle control, (F) Male OC-10 mg/kg, (G) Male OC-250 mg/kg, (H) Male OC-500 mg/kg representative sections.
